# Supplementary material for: Acidosis-Induced TGF-β2 Production Promotes Lipid Droplet Formation in Dendritic Cells and Alters Their Potential to Support Anti-Mesothelioma T Cell Response
Source: Cancers (Basel). 2020 May 19;12(5):1284. doi: 10.3390/cancers12051284 (PMC7281762; doi:10.3390/cancers12051284)
Supplement: Supplementary file 1 [file cancers-12-01284-s001.docx]

**SUPPLEMENTARY FIGURES.**

**
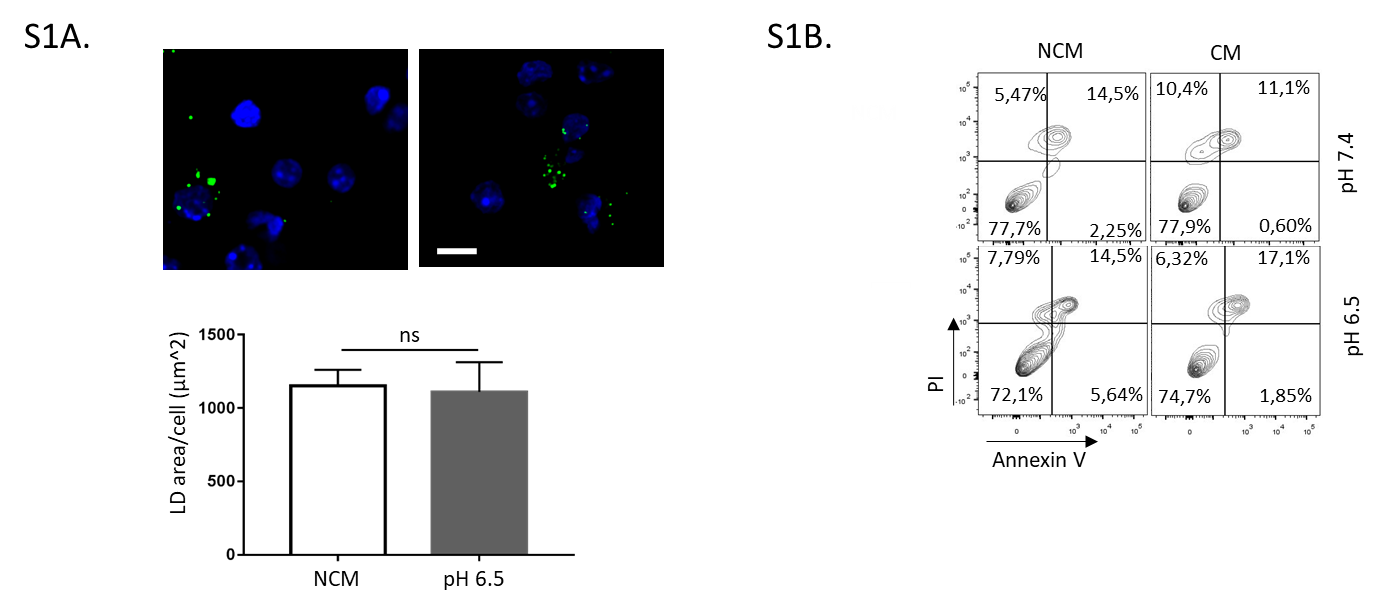
**

**Figure S1. Acidic pH *per se* does not promote LD accumulation in dendritic cells and does not significantly influence cell survival. A**. DC were incubated in control medium (pH 7.4) or pH6.5-buffered (unconditioned) medium for two days. Representative pictures of LD content as determined using BODIPY 493/503 staining are shown together with quantification of the cellular area covered by LD (n = 3, ns = non-significant). **B.** DC were incubated in unconditioned media (NCM) or conditioned media (CM) from AE17 mesothelioma cells at the indicated pH and the extent of cell death was measured after two days using Annexin V and PI staining. The data presented in this figure are representative of three different experiments.

**
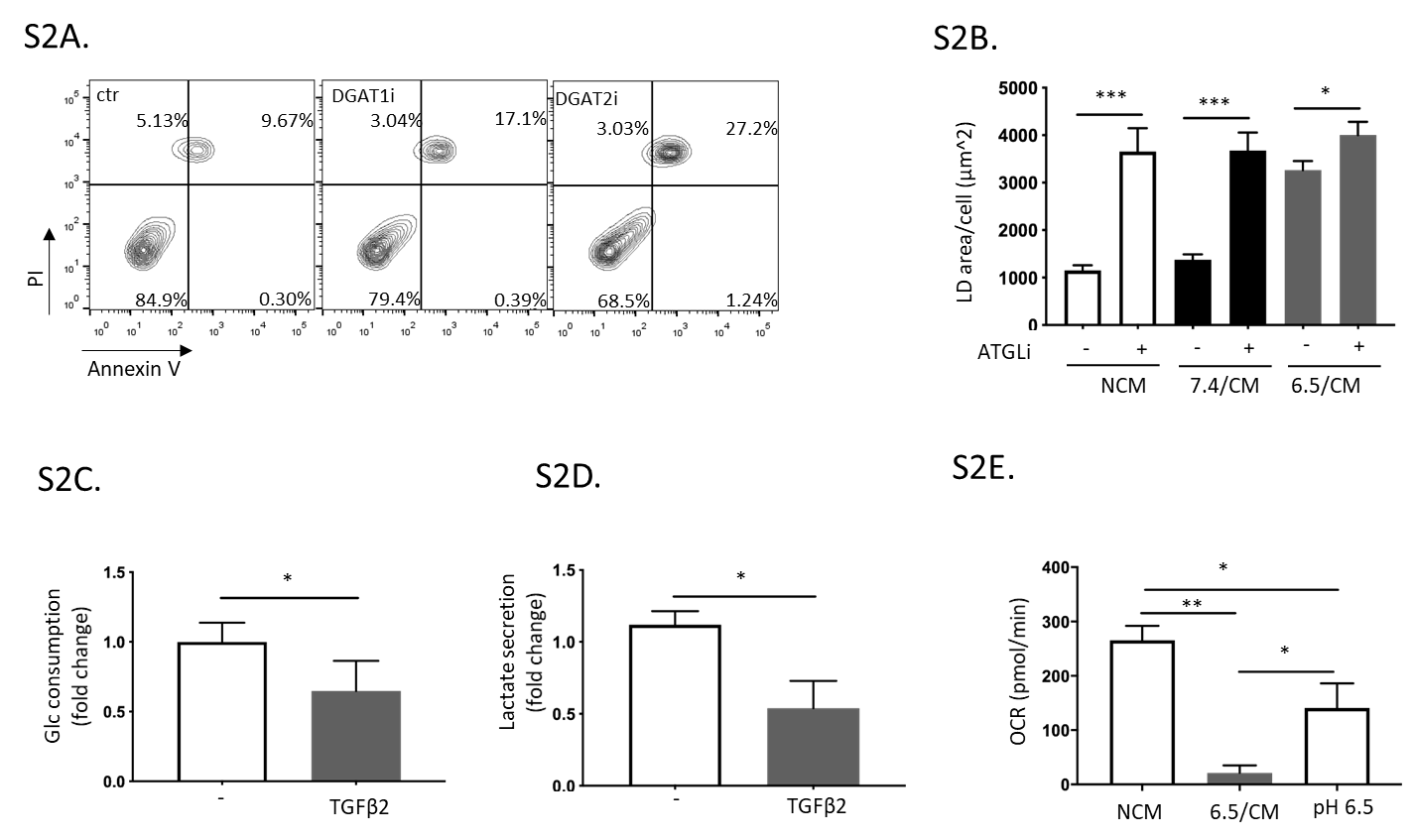
**

**Figure S2. Acid-driven TGF-β2-mediated LD accumulation influences DC metabolism. A.** The extent of DC death after 2 days in 6.5/CM in the presence of either 15 µM A922500 (DGAT1i) or 10 µM PF-06424439 (DGAT2i) was measured using Annexin V and PI staining. The data presented in this figure are representative of three different experiments. **B.** Effect of 10 µM Atglistatin (ATGLi) on cellular LD content (as determined using BODIPY 493/503) in DC incubated for 2 days in the presence of non-conditioned medium (NCM) or conditioned medium from Ab1 mesothelioma cells maintained at pH7.4 or pH6.5 (7.4/CM and 6.5/CM, respectively) (n=3, *p<0.05, ***p<0.001). **C-D.** Effect of 4 ng/ml recombinant TGF-β2 (2 days exposure) on glucose consumption (**C**) and lactate secretion (**D**) (n=3, *p<0.05). **E.** Effects of 6.5/CM and pH6.5-buffered (unconditioned) medium on oxygen consumption rate (OCR), as detected using Seahorse XF Analyzer (n=3, *p<0.05, **p<0.01).

**
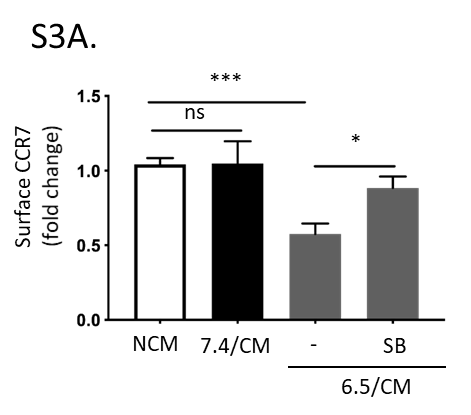
**

**Figure S3. DC exposed to acidic mesothelioma milieu exhibit reduced migrat­­­­ory potential *in vitro.* S3A.** BMDC were incubated with non-conditioned medium (NCM) or treated for 2 days either with conditioned medium from AE17 mesothelioma cells maintained at pH 7.4 or pH 6.5 (7.4/CM and 6.5/CM, respectively). **A.** Effects of of 6.5/CM with or without 5 µM SB-431542 on CCR7 surface expression as determined by flow cytometry (n=3, *p<0.05, ***p<0.001, ns = non-significant).

**
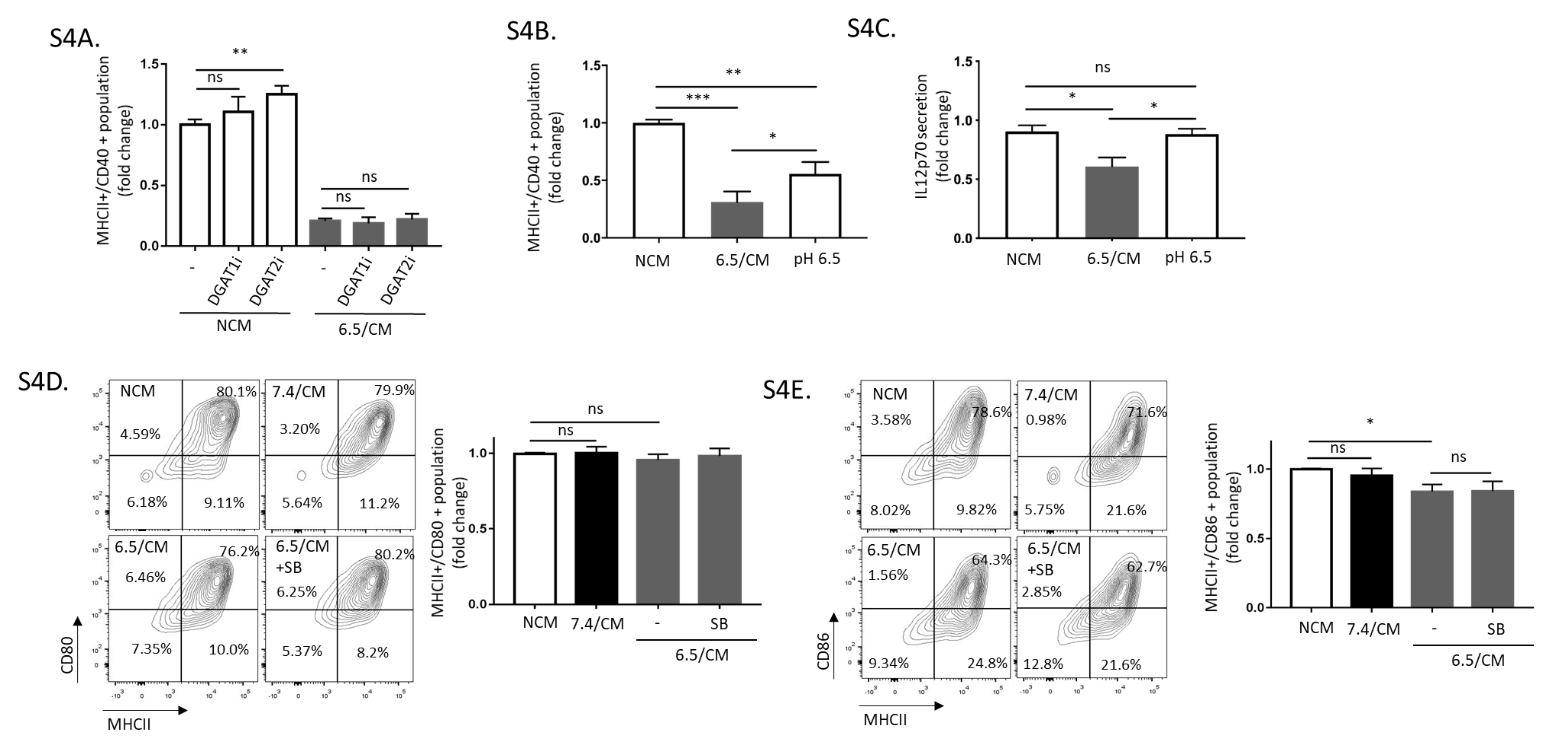
**

**
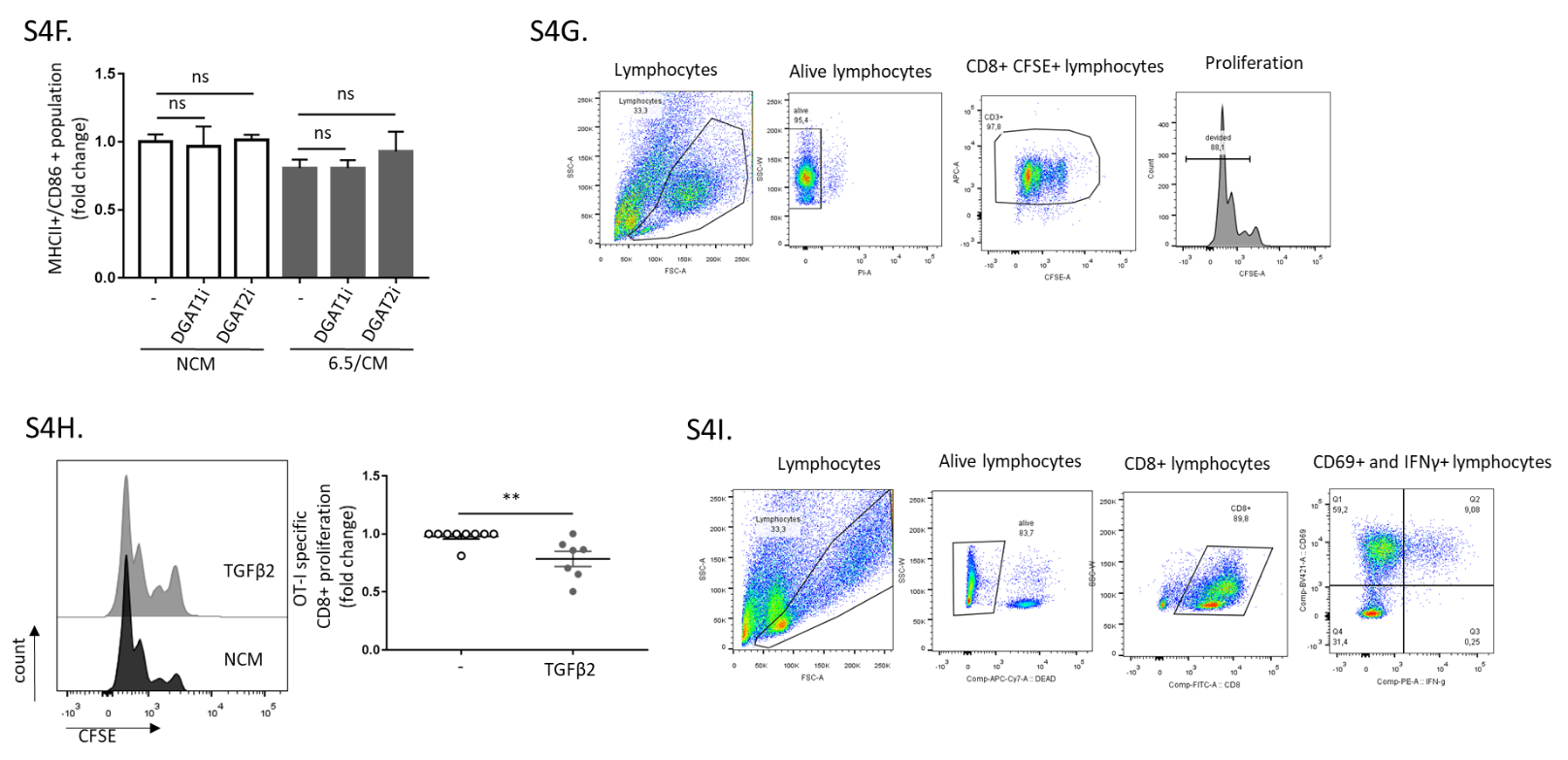
**

**Figure S4.** **Acidic mesothelioma milieu alters DC phenotype.** BMDC were incubated with non-conditioned medium (NCM) or treated for 2 days either with conditioned medium from AE17 or Ab1 mesothelioma cells maintained at pH 7.4 or pH 6.5 (7.4/CM and 6.5/CM, respectively). **A.** Effects of either 15 µM A922500 (DGAT1i) or 10 µM PF-06424439 (DGAT2i) on surface expression of MHCII^+^/CD40^+^, as determined by flow cytometry. **B-C.** Effects of 6.5/CM and pH6.5-buffered (non-conditioned) medium on surface expression of MHCII^+^/CD40^+^ (**B**) and IL-12p70 secretion as detected by ELISA (**C**). **D-E.** Effects of 6.5/CM with or without 5 µM SB-431542 on MHCII^+^/CD80^+^ (**D**) and MHCII^+^/CD86^+^  (**E**). **F.** Effects of 6.5/CM with either 15 µM A922500 (DGAT1i) or 10 µM PF-06424439 (DGAT2i) on MHCII^+^/CD86^+^ expression. **G.** Gating strategy for the detection of CFSE-based proliferation.  **H.** Effects of 4 ng/ ml recombinant TFG-β2 on OT-I specific CD8^+^ proliferation as measured using CFSE dilution. **I.** Gating strategy for the detection of INF-γ^+^/CD69^+^ CD8^+^ population. The charts and histograms presented in this figure are representative of at least three different experiments, *p<0.05, **p<0.01, ***p<0.001; ns = non-significant.

**
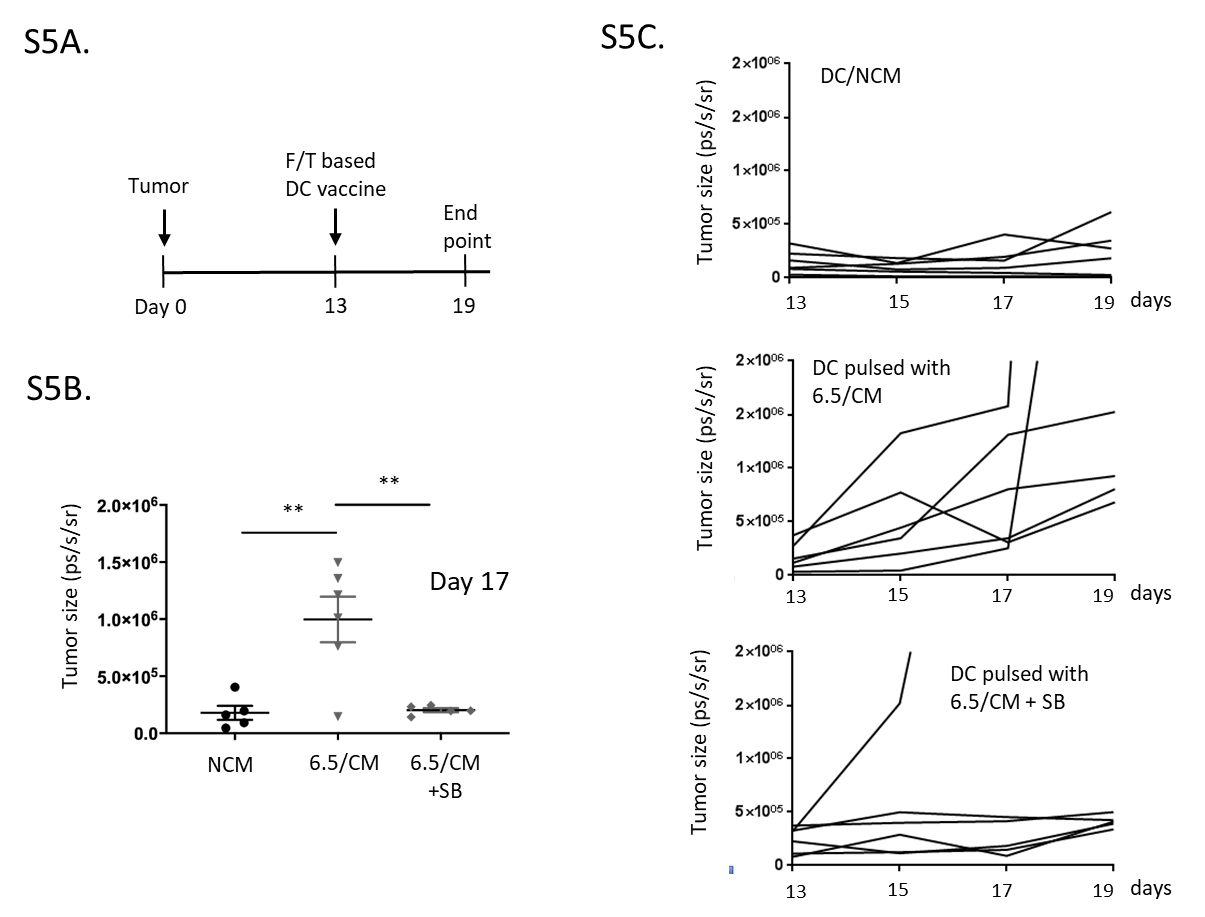
**

**Figure S5.** **Acidic mesothelioma milieu alters the therapeutic efficacy of DC-based vaccination.** Balb/c mice were injected i.p. with 1x10^5^ Ab1-luc and at day 13 were vaccinated with DC pulsed with mesothelioma cell lysates in the presence of either non-conditionned medium (NCM), 6.5/CM or 6.5/CM + 5 µM SB-431542. **A.** Protocol of autologous DC vaccination regimen. **B.** Tumor sizes determined from bioluminescence signal measurements at day 17 for each condition (n=6 mice per group, **p<0.01). **C.** Time course of peritoneal mesothelioma growth determined from bioluminescence signal measurements (n=6 mice per group).

**
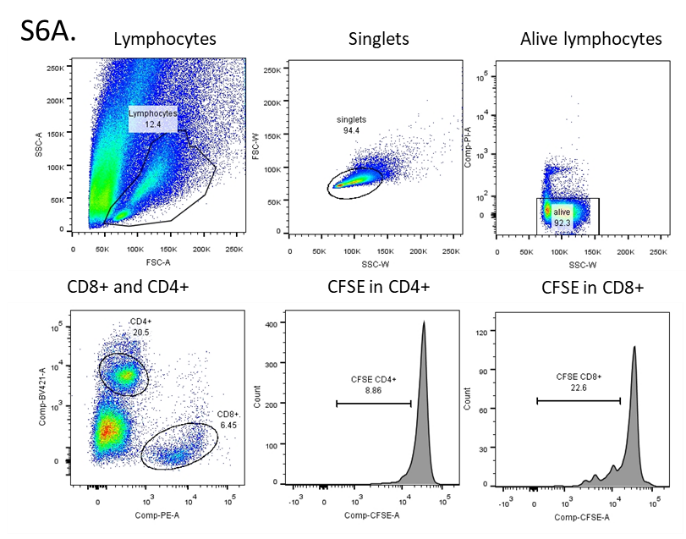
**

**
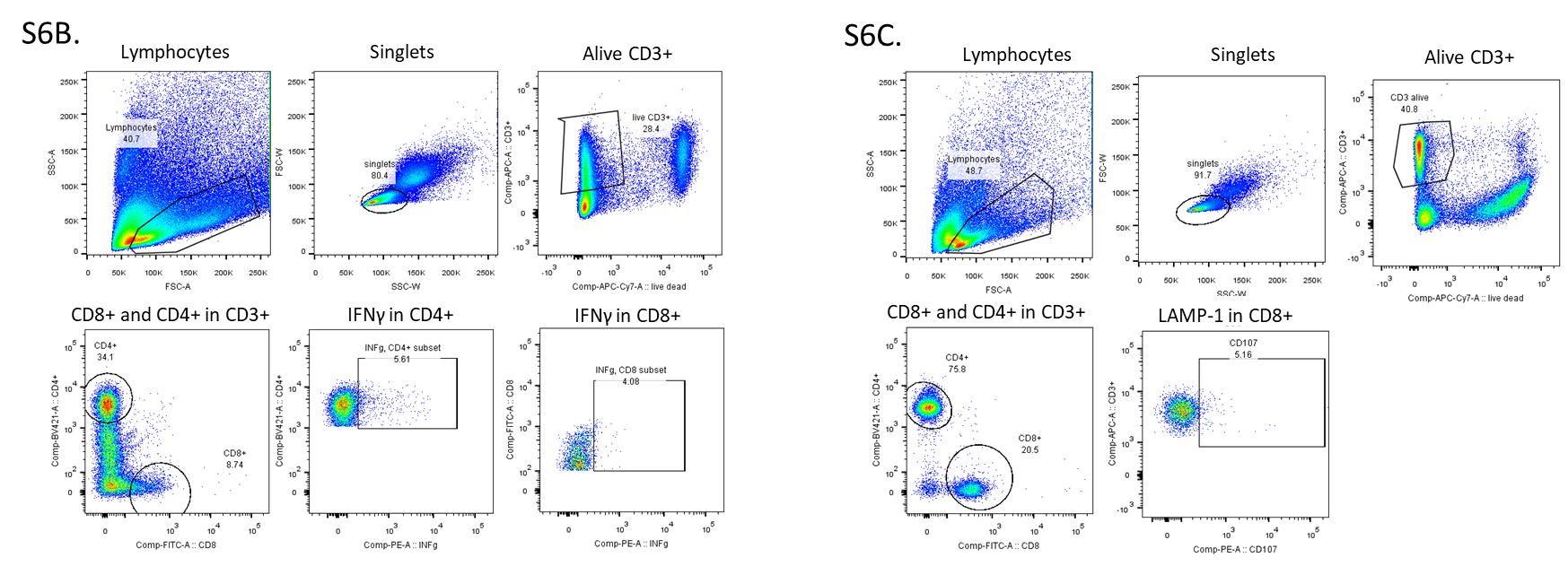
**

**
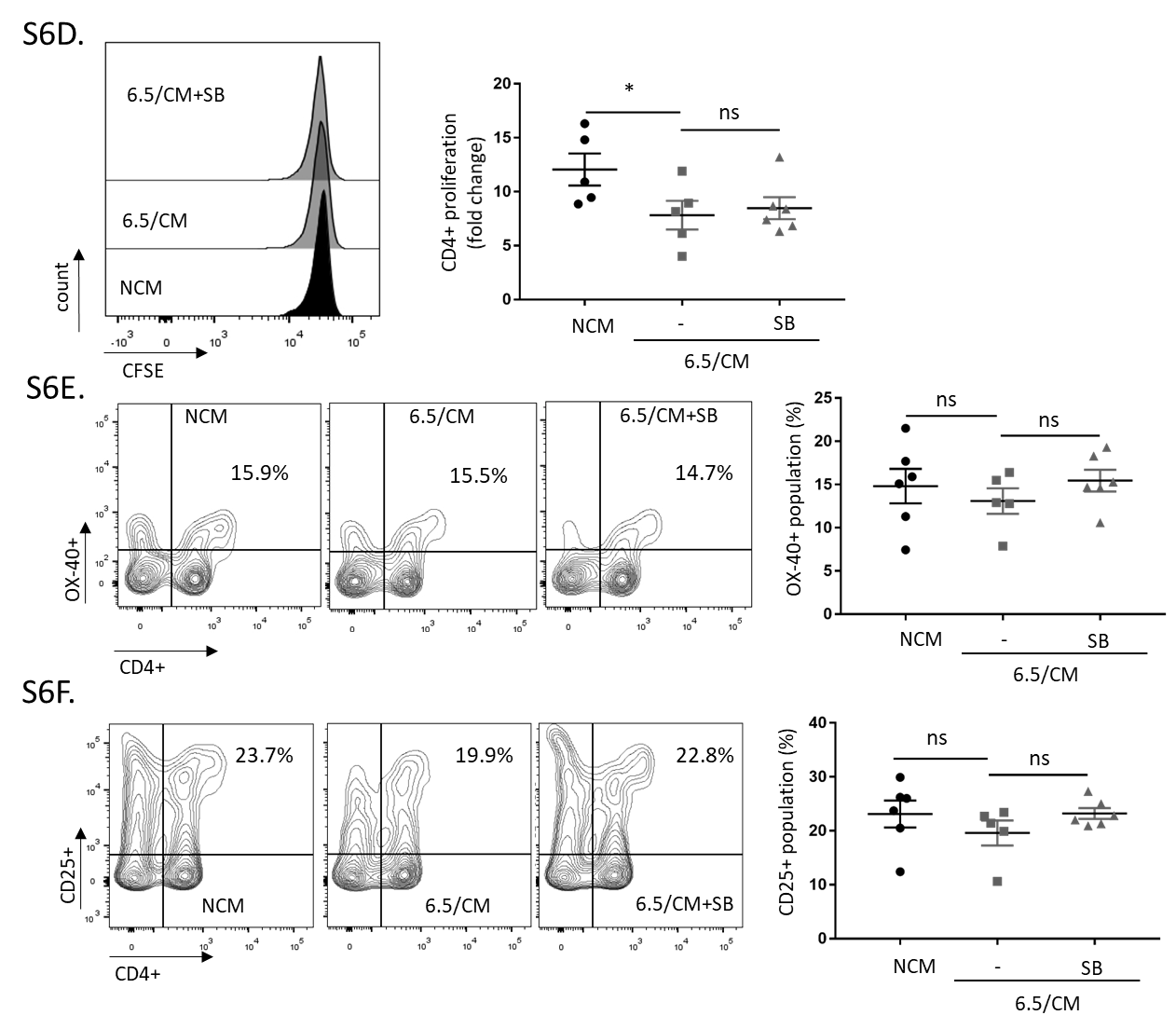
**

**Figure S6.** **Acidic mesothelioma milieu marginally alters the ability of DC-based vaccine to induce CD4^+^ T cell response *in vivo.* A-C**. Gating strategy for CFSE-based CD4^+^ and CD8^+^ proliferation (**A**), INF-γ production (**B**) and LAMP-1 expression (**C**). **D-F.** CD4^+^ splenocytes collected at day 14 post-vaccination (i.e. NCM, 6.5/CM and 6.5/CM + 5 µM SB-431542, see Figure 5) were re-exposed to the Ab1 mesothelioma cells. Proliferation of CD4^+^ splenocytes was detected based on CFSE dilution (n=6, *p<0.05, ns = non-significant) (**D**) and activation was probed based on OX-40 (**E**) and CD25 surface expressions (**F**) (n=6, ns = non-significant).
